# Diagnosis and case finding according to key partner risk populations of people living with HIV in Nigeria: A retrospective analysis of community-led index partner testing services

**DOI:** 10.1016/j.eclinm.2021.101265

**Published:** 2022-01-04

**Authors:** Amobi Onovo, Abiye Kalaiwo, Angela Agweye, Godwin Emmanuel, Olivia Keiser

**Affiliations:** a Office of HIV/AIDS and TB, US Agency for International Development, Nigeria; b Heartland Alliance Nigeria LTD, Nigeria; c Institute of Global Health, University of Geneva, Switzerland

## Abstract

**Background:**

The HIV epidemic in Nigeria is concentrated in Key Populations (KP), people who inject drugs (PWID), men who have sex with men (MSM), female sex workers (FSW), and partners of people living with HIV. Due to stigma and discrimination, these groups have low access to HIV testing services (HTS) and linkage to treatment is challenging. To address this gap, index partner testing, targeting sexual contacts and injecting partners of KP index clients, was introduced in 2017.

**Methods:**

The study was a retrospective analysis of community-led HIV index partner testing-involving review of secondary data from partner notification services registers. Between October 1, 2018, and September 30, 2019, HIV testing as part of index partner testing services was offered at nightclubs, hotels, and community-based ART clinics in the states of Akwa Ibom, Cross River, and Lagos. Index testing was assisted by peer navigators. We used provider and passive Partner Notification (PN) methods. In-person and social network methods were used to recruit partners of KP. We described the implementation of index partner testing services as part of the national KP program, analyzed PN delivery models, and calculated HIV seropositivity among persons who underwent Index Partner Testing. One-Way ANOVA and Tukey-HSD test were performed to determine whether the differences in mean HIV seropositivity between partners are statistically significant.

**Findings:**

PN was predominantly done through provider referral 5,159 (68.3%) and passive/client referral 2,278 (30.1%). A total of 3,119 index partners; 1,322 FSW (42.4%), 1,255 MSM (40.2%) and 542 PWID (17.4%) identified 8,989 sexual and injecting partners (index partner ratio 1:2.9). Among the partners, 7,556 (84.1%) were first-time testers, and 79.4% (5,999) of male partners tested. Of the 3,753 (49.7%) partners who tested HIV-positive, 3,492 (93.0%) were enrolled in HIV care. HIV seropositivity rate was 65.5% (1,021/1,557) among females and 45.5% (2,732/5,999) among males and was disproportionately higher among PWID injecting partners 99.1% (581/586), PWID sexual partners 98.9% (433/438) and MSM sexual partners 95.6% (605/633) in Cross river compared with 71.4% (575/805) in FSW sexual partners.

**Interpretations:**

Including index partner testing as part of a community-led HTS can help improve HIV case-finding approach for KP, particularly for reaching first-time testers, male KP, and persons not yet diagnosed with HIV. Scale-up of index partner testing within community-led HTS is essential for achieving the United Nations 95–95–95 goals.

**Funding:**

This research received no specific grant from any funding agency in the public, commercial or not-for-profit sectors.

**Research in context**

**Evidence before this study**

Recent systematic reviews show that KP's low uptake of HTS is often due to hostile services, fear of stigma, discrimination, and punitive laws and practices that criminalize behaviors and, as a result, limit access to health care, including HTS. On November 3, 2020, we searched Google scholar, PubMed and the American Medical Association website for published research articles using the terms "Partner Notification Services," "Index Testing," "KP sexual contacts," and "KP social network," with no language or date restrictions. Our search turned up descriptive, retrospective, systematic review, randomized controlled trials and meta-analysis papers, that suggest that testing of biological children and sexual partners utilizing HIV index testing model can be viable to identify and link children, adolescences and adult into care and treatment.

**Added value of this study**

The authors developed a flowchart of the HIV index partner testing model among KP, examined partner notification (PN) delivery techniques, and calculated HIV seropositivity among persons who underwent Index partner testing among Key populations in three Nigerian states. Our study analyzed HIV PN delivery models selected for 7559 sexual contacts plus injecting partners of 3119 KP index clients (index partner ratio 1:2.9) who accepted index partner testing. Study showed high uptake of the provider assisted notification and referral process for 68.3% index partner testers compared to the other PN methods.

**Implications of all the available evidence**

Including index partner testing as part of a community-led HTS can help improve HIV case-finding approach for KP, particularly for reaching first-time testers, male KP, and persons not yet diagnosed with HIV. Results suggest improvements in the rate of HIV testing among males through HIV partner notification services. The high enrolment in care and treatment services can be attributed to a more personal engagement of sexual and injecting partners by HTS peer-outreach workers at the community level.

Alt-text: Unlabelled box

## Introduction

People's knowledge of their own, and their partner's, human immunodeficiency virus (HIV) status is essential to the success of the global HIV response. The overarching goals of providing HIV testing services (HTS) are to deliver a diagnosis and effectively facilitate access to and uptake of HIV prevention, treatment, and care services. Over the past decade, low- and middle-income countries in Africa recorded substantial improvements in the percent of people living with HIV who are aware of their HIV status from 10% in 2005 to 50% in 2015.^1^ These achievements have been possible largely because of the scale-up and use of the wide availability of low-cost rapid diagnostic tests. The term antiretroviral therapy (ART) is referred to as treatment with drugs that inhibit the ability of the HIV or other types of retroviruses to multiply in the body. The growing availability and use of rapid diagnostic tests have made it possible to increase task sharing. This has enabled HTS to also be delivered by trained lay providers and to be implemented in more targeted locations, ranging from routine testing in facilities to community-based outreach. Despite these achievements, a substantial testing gap remains. According to recent estimates, 77% of all people diagnosed with HIV are on ART; however, 40% of all people with HIV remain undiagnosed.^1^ Furthermore, despite the annual increases in HIV tests and HIV testing coverage,^2^ in many settings, HTS is not sufficiently focused. Many of those at highest risk, such as partners of people with HIV, young people in high HIV prevalence settings, and key populations (KP) worldwide, remain unreached.

KP such as female sex workers (FSW), men who have sex with men (MSM), and people who inject drugs (PWID) are disproportionately affected by HIV. They comprise approximately 36% of the 1.9 million new adult HIV infections that occur each year.^1^^,^^3^ In 2020, Key populations, their clients, and sexual partners accounted for 64% of new HIV infections in West and Central Africa, and 25% of new HIV infections in the East and Southern African subregion.^4^ Data from Senegal, The Gambia, Côte d'Ivoire, Ghana, and Nigeria indicate that a substantial number of infections occur^5^, ^6^, ^7^, ^8^ among MSM, many of whom also report having sex with women.^9^ Based on the 2018 national KP size estimation carried out by National AIDS, Sexual Transmitted Infections Control and Hepatitis Program (NASCP),^10^ the total KP in Nigeria is estimated to be 720,000. About three-quarters (69%) of the KP have been tested for HIV under the national KP program.^11^ In Nigeria, the prevalence of HIV is on the decline, it dropped from 5.8% in 2001 (ANC Survey report) to 1.5% in 2018 Nigeria AIDS Indicator and Impact Survey (NAIIS). However, KP, including FSW, MSM, and PWID continue to have a high HIV prevalence. HIV prevalence is respectively 22.9%, 19.4%, and 8.6% in MSM, brothel-based female sex workers, and non-brothel-based female sex workers.^12^ In Nigeria, ART coverage in KP is unknown.^13^, ^14^, ^15^ KP is understudied and likely underserved resulting in a limited characterization of their HIV prevention, treatment, and care needs.^14^

Although countries are increasing including key populations in their national HTS guidelines, implementation remains limited, and testing coverage continues to be low in most settings.^2^ Poor coverage and low uptake of HTS among KP are not only related to availability but also the acceptability of services. Low acceptability frequently reflects unfriendly services, fear of stigma, discrimination, and punitive laws and practices that criminalize behaviors and, thereby, discourage access to health services, including HTS.^3^ These challenges require a new focus and new approaches to reach people with undiagnosed HIV infection. Many countries and programs are exploring innovative approaches, such as index partner testing or partner notification services to delivering HTS to achieve national and global testing targets.

Index partner testing can be defined as an approach by which a person with confirmed HIV infection (index case) is asked to contact family members (children, spouse, sexual partners, siblings, and parents) to see if they will accept an HIV test.^4^ The approach has proven to be a key intervention in diagnosing people living with HIV (PLHIV) and enrolling and retaining them in care and ART.^16^ To increase HIV case identification among KP, the national KP program funded by the President's Emergency Plan for AIDS Relief (PEPFAR) and implemented through Non-Governmental Organizations (NGOs) in Nigeria introduced index partner testing in October 2017. All KP patients accessing ART were encouraged to bring their family members, spouses, sexual contacts, and/or injecting partners for HIV testing. The national KP program engages directly with KP and delivers client-centered services through community-led programming. HIV testing, prevention, and care services are provided to KP through differentiated service delivery methods, with services delivered in areas where KP may be served without discrimination. To build access to services, the national program supports drop-in centers and personalized outreach, and alternative pick-up points, such as one-stop-shops that provide community-based treatment initiation and refills in addition to testing services.

We aim to describe the implementation of index partner testing as part of the national KP program and to present the first results. We established the HIV seropositivity rate among male and female sexual contacts plus injecting partners of KP index clients and analyzed HIV partner notification services and referral services of HIV positive index clients.

## Methods

### Study design and setting

The study was a retrospective analysis of community-led HIV index partner testing-involving review of secondary data from partner notification services registers. The registers documented HIV-positive index clients who were offered partner notification services, and the sexual and injecting partners elicited. All clients offered partner notification services in the three states were included in this study. Nigeria is organized into 36 federating states and the Federal Capital Territory which hosts the national government. The states are further sub-divided into 774 Local Government Areas (LGA). The cross-sectional retrospective study was conducted in three focus states Akwa Ibom, Cross River, and Lagos. Under the national KP program, these states were prioritized for prevention and comprehensive HIV/AIDS treatment interventions due to the high population density and the high number of PLHIV. The Nigerian HIV/AIDS Indicator and Impact Survey has shown that Akwa Ibom has the highest prevalence rate of HIV in the country”. The states of Cross River and Lagos were rated 10th and 14th in terms of HIV prevalence rates, respectively”.^17^ Supplementary 1 is a map of Nigeria that depicts the study regions and the distribution of PLHIV throughout the country based on the 2019 UNAIDS SPECTRUM HIV estimates. Across several sub-Saharan Africa countries (including Nigeria), UNAIDS currently uses routine program data in Spectrum software to estimate the national HIV prevalence and PLHIV burden. The Spectrum package uses the HIV prevalence over time from routine program data or survey data together with demographic information and epidemiological assumptions to model age-specific HIV prevalence, and incidence and mortality rates, and the total number of people living with HIV.^18^ In 2017, the national HIV program implemented partner HIV notification services. The primary source of data for this study came from Heartland Alliance Nigeria's PN activities, which took place between October 1, 2018, and September 30, 2019. The 12-month timeframe corresponds to the program cycle timeline or fiscal year in which program interventions are executed in Nigeria with technical and financial assistance from PEPFAR. The national KP program consists of an integrated HIV prevention and treatment program that identifies HIV-positive KP in the community and links them to care and treatment at the LGA level.^19^

### Definitions of HIV partner notification delivery models

In this study, partner notification was defined as a voluntary process whereby a trained provider asks clients diagnosed with HIV about their sexual partners and/or drug-injecting partners, and then, if the HIV-positive client agrees, the trained provider offers these partners HTS. In this study, partner notification was done in two ways: passive and assisted.^20^ Under the passive HIV partner notification services HIV-positive clients were encouraged by trained providers to disclose their HIV status to their sexual and/or drug-injecting partners and to suggest HTS to the partner(s) given their potential exposure to HIV infection. Under the assisted HIV partner notification services, consenting HIV-positive clients were assisted by a trained provider to disclose their status or anonymously notified their sexual and/or drug-injecting partner(s) of their potential exposure to HIV infection. The trained provider offered HIV testing to these partner(s). Assisted partner notification was done using contract referral, provider referral, or dual referral approaches.

Contract referral: HIV-positive clients entered a contract with the trained care providers and agreed to disclose their status and the potential HIV exposure to their partner(s) by themselves. According to the contract, HIV-positive clients are expected to refer their partner(s) to HTS within a specific time. If the partner(s) of the HIV-positive individual does not access HTS or contact the health provider within that period, then the providers contact the partner(s) directly and offered voluntary HTS.

Provider referral: With the consent of the HIV-positive clients, the trained provider confidentially contacted the person's partner(s) directly and offers the partner(s) voluntary HTS.

Dual referral: The trained provider accompanied and provided support to HIV-positive clients that disclosed their status and the potential exposure to HIV infection to their partner(s). The care provider offered the partner HTS voluntarily (s). Anonymous techniques in which the index case and provider notified the partners of potential exposure without identifying the index partner's position or engagement

### Index testing services among key populations in Nigeria

The national KP program consists of an integrated HIV prevention and treatment program that identifies HIV-positive KP in the community and links them to care and treatment at the local government area or community level. Index testing is also referred to as partner testing/PN services. In this approach exposed contacts (i.e., current, or past sexual partners, and anyone with whom a needle was shared) of an HIV-positive person (i.e., index client), were offered HIV index testing. Every newly diagnosed HIV-seropositive becomes an indexing client from whom to elicit contacts. All index testing services meet WHO's 5C minimum standards, including consent, counseling, confidentiality, correct test results, and connection to HIV prevention (for HIV-positive and HIV-negative individuals), and linkage to HIV care and treatment for HIV-positive individuals. Specific program guidelines, tools, and standard operating procedures were utilized to provide safe and ethical index testing services, in compliance with the WHO consolidated guideline on partner notification services. To reduce the risk of harm, trained providers ensured that all index clients got counseling and support services, such as helplines and screening for Intimate Partner Violence (IPV), as per WHO standards. To ensure compliance with the index testing guidelines, community-based organizations, civil society groups, and networks of key populations conducted community-led monitoring activities (gather quantitative and qualitative data about HIV services). HIV-positive clients were offered multiple options for assisted partner notification, and the approach selected was based on client preferences. Exposed contacts were traced and offered HIV testing.

The national KP program is entirely community-based, and HIV testing as part of index partner testing services was offered in nightclubs, hotels, drop-in-centers, or community-based ART clinics (One-Stop-Shops (OSSs)). Nightclubs and hotels are ideal settings for HTS interventions, because KP members visit these venues to socialize, seek clients, or participate in key population–defining behaviors. In these areas, HIV testing was primarily based on the desire or request of the index sexual and/or injecting drug use partners. HIV prevention programs for KP are provided through drop-in centers, which are safe places in the community. OSS model for KP established safe spaces in the communities for biological (e.g., HIV testing, sexual transmission infection (STI) screening), behavioral (e.g., risk assessment, peer education) and structural (e.g., income generating activity) interventions. It integrates differentiated strategies that optimize efficiency along the 95–95–95 cascade.^21^ The project developed standard operating procedures and data-collection tools (client intake forms, HIV Index Testing Services register, or logbook) for use by providers during counseling of HIV index patients and meeting their relatives. For contact to be counted under the index testing program, he/she must be tested for HIV and receive their result or be known as HIV positive. The contact could either self-report a known exposure to someone with HIV as their reason for testing, have an index testing referral letter/card/coupon given to them from their HIV-positive partner/family member (client-referral approach), or have been identified during the elicitation process and contacted through a provider referral. To reduce the double-counting of index partners and account for re-testers in a reporting period, tracking systems with “unique identifiers” were established and used to monitor the frequency of contact/outreach of a single index case over time. A unique ID was generated and assigned to an index partner before HIV testing commences, and the results collected were entered into an electronic database. The partner elicitation process of index testing is a continuous process. Peer navigators followed standard operating procedures to determine when KP was asked again about any new partners or previous partners that may not have been disclosed previously. Index testing was assisted by peer navigators. Peer navigators are KP who were recruited and trained as peer-outreach workers to increase demand for tailored HIV services, improve the quality of behavior change communication and increase access to HIV testing.

HIV testing delivery services to KP in the target communities adopted a 2 step HIV rapid testing– serial algorithm testing strategy. The fourth-generation rapid test kits were used for HIV testing (D*etermine*™ *HIV-1/2 Ag/Ab Combo*). A non-reactive HIV test result after the first test was considered as HIV-negative. A reactive result was tested with a second test. When the result was reactive again, it was considered HIV-positive and when it was non-reactive it is considered HIV-negative. HIV testing and counseling, and requests for elicitation of sexual and injecting partners all take place in a private and confidential environment. HIV testing and counseling are available to sexual partners at convenient hours (including nights and weekends) and places (home, facility, or other community locations). HIV self-test kits are also provided to clients, which they can distribute to their sexual partners. Clients who rejected HIV testing services were offered the opportunity to be contacted again after a set length of time. Preventive treatments, such as harm reduction interventions, behavioral interventions, sexual and reproductive health interventions, pre-exposure prophylaxis (PrEP), information on post-exposure prophylaxis, condoms, and lubricants, are offered for sexual and injecting contacts of clients who test negative. To support HIV treatment and care for HIV positive KP, the national program supports drop-in centers, personalized outreach, and alternative pick-up locations, such as one-stop-shops that offer community-based treatment initiation and refills in addition to testing services. Multi-month dispensing for both PrEP and ART are provided and dispensed through community-based clinics or drop-in centers.

### Implementation of index testing services

Index testing cascade for KP is categorized into four specific steps based on four program performance indicators. These steps are part of a cascade of implementation that begins with an offer of index testing services to the index client (newly diagnosed KP) and ends in provision of an HIV test to the contacts named by the index client. The steps are:

(1) Number of index clients who were offered index testing services. This is the number of index clients (newly diagnosed positive KP) who were offered index testing services (regardless of whether those services were accepted by the index client).
(2) Number of index clients who accepted index testing services. This is the number of index clients who accepted provision of index testing services by a provider (including client providing informed consent, acceptance of counseling on index testing, acceptance of elicitation of current or past sexual or injecting partners and/or partner notification).
(3) Number of contacts provided by the index client. This is the number of contacts provided by the index client because of accepting index testing services.
(4) Number of contacts who were tested for HIV. This is the number of contacts who were tested for HIV and received their results. Previous or known positives are also recorded.

### Study population

All contacts (sexual contacts or injecting drug users) of HIV-positive FSW, MSM, and PWID aged ≥15 years who received partner notification services were included in the analysis. For simplicity, we refer to them as “partners” in the rest of the text. The index case's spouse and casual sex partners are the sexual partners included in the study. According to the national KP program, children under the age of 15 are not classified or named in any of the KP categories and hence were not included in this study. Only adult KP is enrolled in the care and treatment program. Children of KP, on the other hand, are linked to HIV prevention and treatment activities through the national HIV program for the general population. In-person and social network-based HIV testing approaches were used to recruit index clients. KP high-risk networks are used in social network strategies to refer people for HIV testing. These strategies, which include an enhanced peer outreach strategy, take advantage of social, sexual, and drug-using relationships or behaviours to reach high-risk and hidden individuals who might benefit from HIV testing. The trained care provider urges KP with HIV or those who are HIV-negative and at continuing risk of HIV to encourage and invite others in their sexual, drug injecting, or social networks to engage in voluntary HTS as part of the social network testing strategy. FSW was defined as women who receive money or goods in exchange for sexual services, and who consciously define those activities as income generating even if they do not consider sex work as their occupation.^22^ MSM was defined as (1) self-identification as male and (2) report of oral or anal sex with a man in the prior 12 months.^23^ PWID was defined as a person who meets one of the following conditions: (1) self-reporting ever injecting any illicit drug and having a visible injection site on the body, (2) self-reporting injecting illicit drugs in the past month.^24^

### Statistical analyses

We analyzed aggregate-level program data from index testing by counting the number of sexual partners plus injecting drug users of HIV positive KP who received HIV index partner testing services between October 1, 2018, and September 30, 2019, at the community level. We conducted four separate analyses: First, we created a flow chart diagram of the HIV index testing model from three Nigerian states. Second, we utilized proportions to characterize the methods for HIV partner notification of index partners' HIV status and calculated HIV seropositivity among those who underwent index testing by state and KP group. Two proportion Z-test using a 5% alpha level (α/2 = 1.96) was used to compare partner HIV seropositivity by states. The null hypothesis (H_0_) for the test is that the HIV seropositivity across KP groups in the study locations is the same. In determining whether to reject the null hypothesis we used the method of identifying rejection regions. We rejected the null hypothesis when the calculated z-score falls into a rejection region. We define the rejection region as an area under a normal standard distribution, where the null hypothesis is not probable. We divided the total number of partners of index clients who tested HIV positive (numerator: previously known HIV positive testers + newly identified HIV positive) by the total number of partners who received HIV testing services to measure the HIV seropositivity. Third, we created a dot plot to examine the differences in HIV testing by state and sex. Fourth, we constructed a violin chart and compared the mean HIV seropositivity across the KP groups using the One-Way Analysis of Variance (ANOVA) test and Tukey's honest significance difference (HSD) test at 95% family-wise confidence level to determine whether the differences between the group means of HIV seropositivity are statistically significant, and to identify the group means that are statistically different from one another. R-software v.4.0.5 was used to perform the analysis. The authors made sure that the manuscript's structure and flow strictly followed STROBE guidelines.

### Ethics

This analysis was conducted with routine data gathered through the national KP program. Informed consent was obtained for all clients who were tested for HIV through index testing in line with the Nigeria HTS policy. Ethical approval was obtained from the Federal Capital Territory, Health Research Ethics Committee, Nigeria (approval no: FHREC/2020/01/123/12–11–20). This study only analyzed anonymized and de-identified data.

### Role of the funding source

This research received no specific grant from any funding agency in the public, commercial or not-for-profit sectors. All the authors accept responsibility for the decision to submit for publication.

## Results

### Overview of HIV index testing cascade

**Figure 1:**
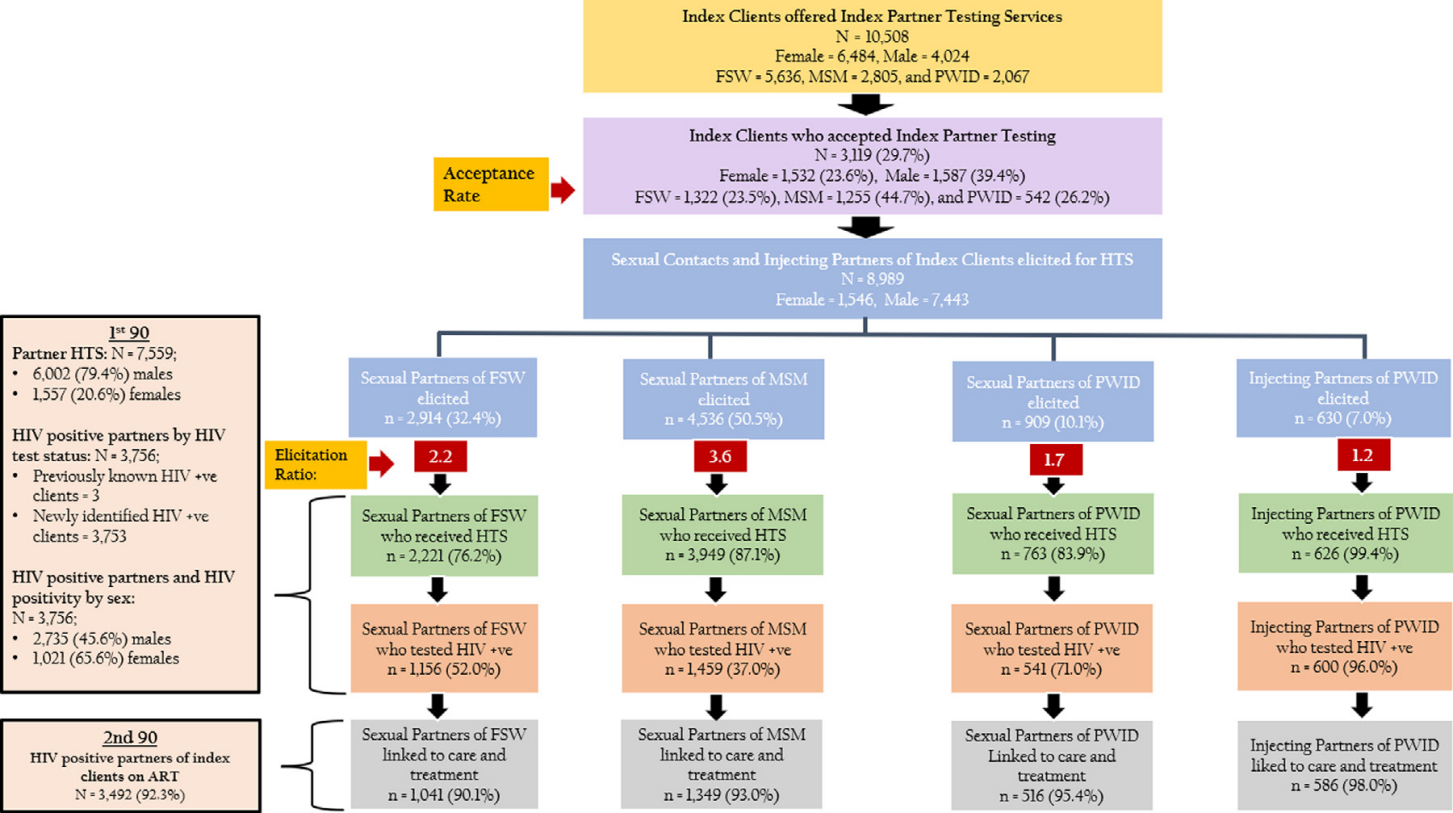
Overview of HIV Testing Services uptake in Index Testing Model from 3 States, Nigeria, October 1, 2018 – September 30, 2019 The flow chart diagram shows how the index partner testing cascade for KP in Nigeria works. The index clients who were offered index partner testing services are shown by the yellow horizontal bar. The purple bar depicts index clients who accepted to participate in index partner testing. The index clients' partners who were elicited for HTS are shown by the blue horizontal bars. The elicitation ratio by KP groups is shown by the red boxes. The light green, orange, and grey horizontal bars show how many partners of index clients were tested for HIV, how many were HIV positive, and how many were on antiretroviral therapy, respectively.

Figure 1 shows the cascade of HIV index testing services and treatment initiation for all KP groups. Of the 10,774 newly diagnosed HIV-positive KP, a total of 10,508 (98%) index clients (5636 FSW, 2805 MSM, and 2067 PWID) were offered index testing services in 54 local government areas across the three states during the study period. Overall, the proportion of index clients who accepted the services was low, 30% (3119/10,508). Of *n* = 3119 KP index clients who accepted the services, acceptance rate was 42.4% (*n* = 1322) for FSW, 40.2% for MSM (*n* = 1255), and 17.4% for PWID (*n* = 542). A total of 8989 partners of the 3119 KP index clients were elicited for index testing services (index partner ratio= 1:2.9; 82.8% (*n* = 7443) male and 17.2% (*n* = 1546) female). HIV testing and counseling uptake ranged from 76.1% (2220/2919) among sexual partners of FSW to 99.4% (626/630) among injecting partners of PWID. Testing uptake was 87.0% (3947/4536) among MSM and 83.9% (763/909) among sexual partners of PWID. Overall, 49.7% (*n* = 3756) were HIV positive. HIV seropositivity rate among partners varied disproportionately. It was 37.0% MSM partners, 52.0% for FSW partners, 71.0% for PWID sexual partners and 96.0% for PWID injecting partners. The results of the two proportional Z-tests revealed that HIV seropositivity was significantly higher in PWID sexual partners than in FSW and MSM partners (z-test = 9.14, *p* < 0.00001 and z-test = 17.39, *p* < 0.00001). HIV seropositivity was also substantially higher in PWID injecting partners than in FSW and MSM partners (z-test = 19.9, *p* < 0.00001 and z-test = 27.5, *p* < 0.00001, respectively). FSW partners similarly had a higher HIV seropositivity than MSM partners (z-test = 11.4, *p* < 0.00001). Linkage to ART was 93.0% (3492/3753) overall and was above 90% in all subgroups.

### Index testing by sex and partner notification delivery models

**Figure 2:**
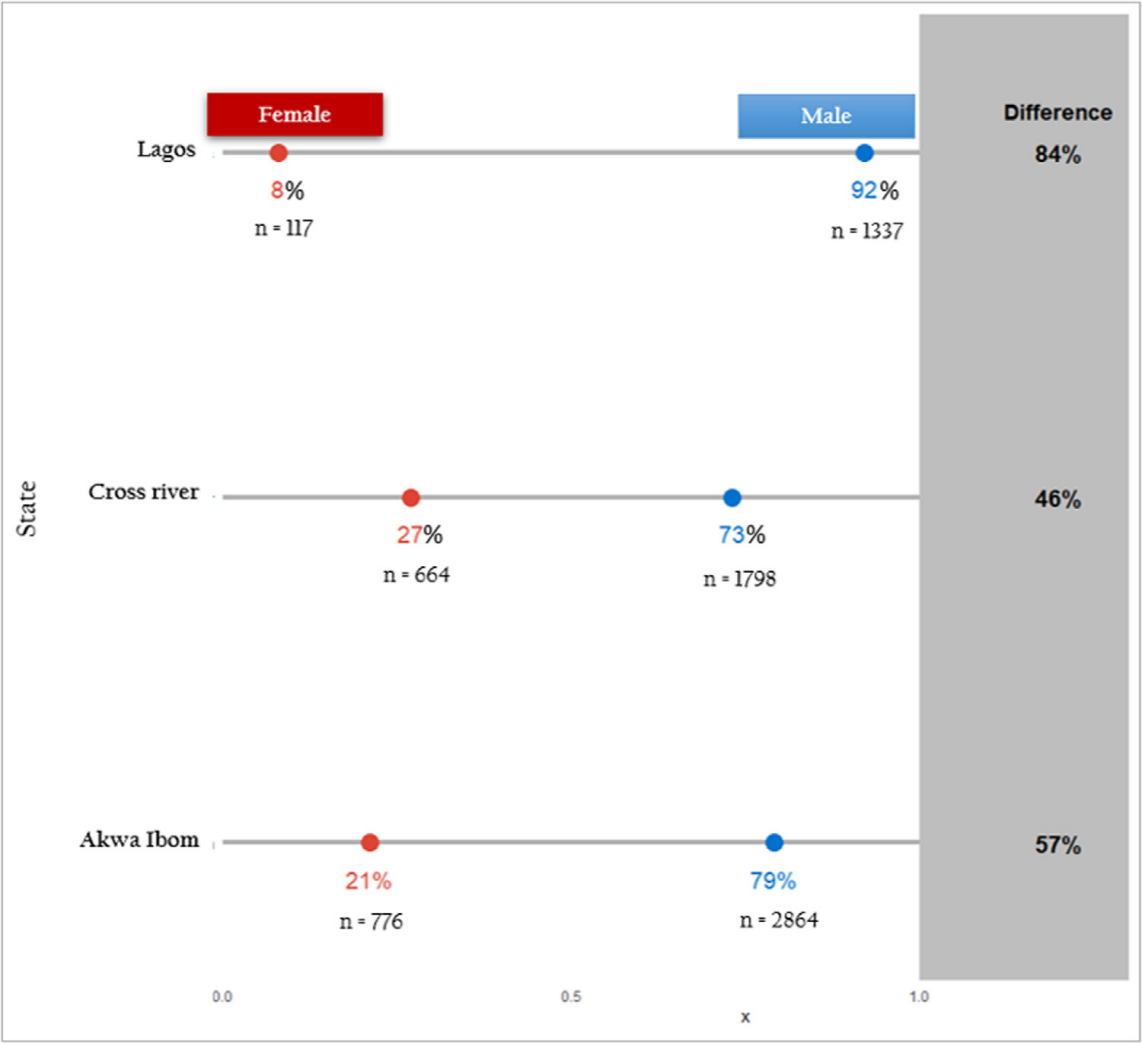
Dot plot showing differences in HIV testing by state and sex, (October 2018 – September 2019) The proportion of females and males who obtained HTS in the index partner testing services is shown by the red and blue dots, respectively. The grey bar depicts the change, and/or difference in HTS distribution by state (vertical axis) and by gender (horizontal axis).

HIV testing services differed considerably by sex. About 79.0% (5999) of males and 21.0% (1546) of female partners elicited for PN services were tested for HIV. Figure 2 indicates that males made up most of the partners who received HTS (Akwa Ibom 78.7%, Cross river 73.0%, and Lagos 92.0%). Almost three-quarters [73.0% (2732/3753)] of those who tested positive were males. The majority 84.1% (7556/8989) partners of KP index partners who received HIV testing and counseling services were first-time testers. KP index clients chose provider referral for 68.3% (5159/7556) of partners, client referral for 30.1% (2278/7556), contract referral for 1.1% (82/7556), and household/dual referral for 0.5% (37/7556) of partners respectively (Table 1).

**Table 1:** Methods for HIV partner notification of index partners HIV status.

Partner Notification method	Sexual Partners of FSW		Sexual Partners of MSM		Sexual Partners of PWID		Injecting Partners of PWID		Overall	
	n	%	n	%	n	%	n	%	n	%
Passive/clients referral	1003	45.2	665	16.8	300	39.3	325	51.9	2281	30.2
Provider referral	1200	54.0	3206	81.2	440	57.7	301	48.1	5159	68.2
Contract referral	14	0.63	46	1.2	22	2.9	0	0.0	82	1.1
Household/Dual referral	4	0.18	32	0.8	1	0.1	0	0.0	37	0.5
Total	2221	100.0	3949	100.0	763	100.0	626	100.0	7559	100.0

### HIV seropositivity rate and linkage to art by state

**Figure 3:**
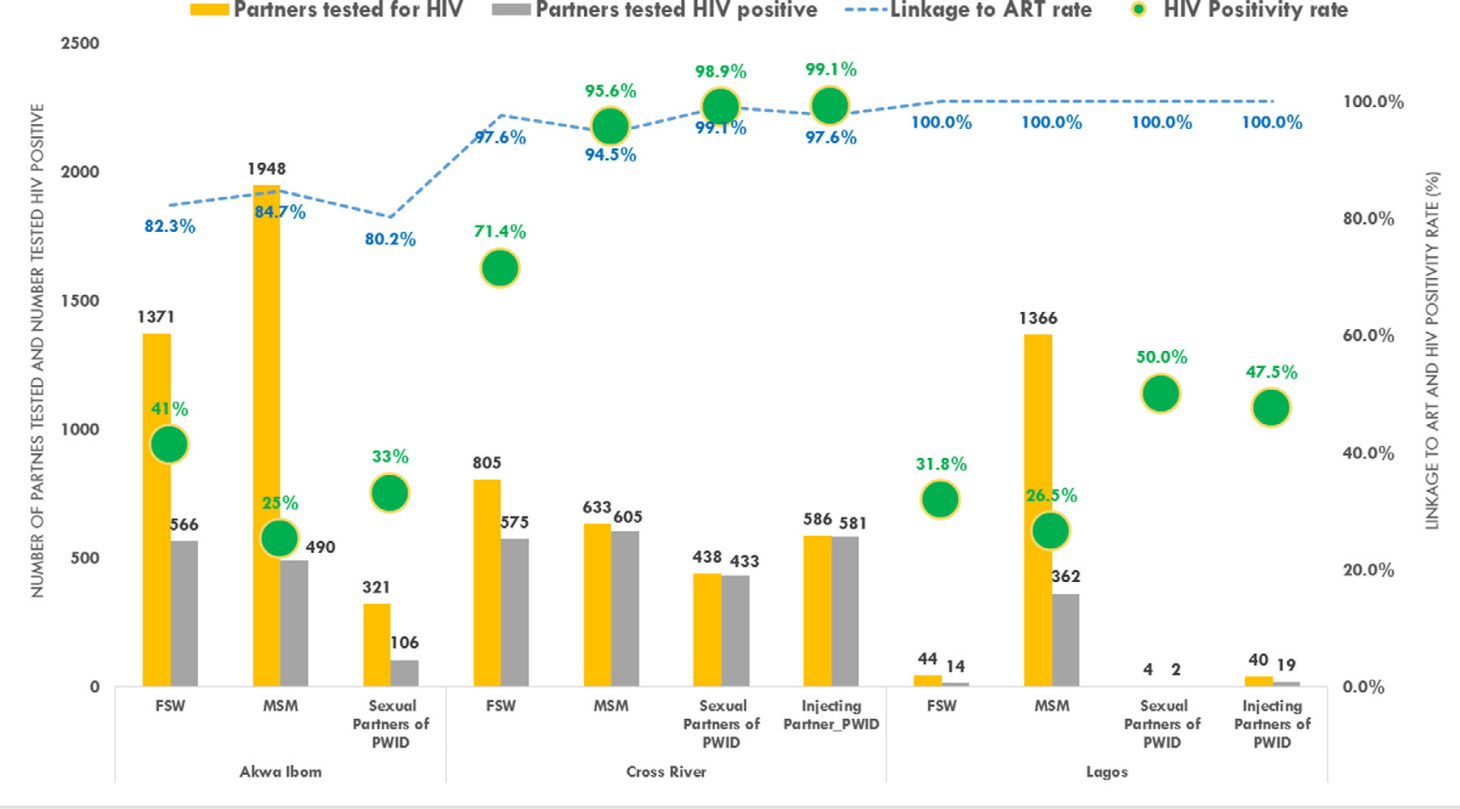
HIV Cascade of tested index partners by key population group and State, October 2018 – September 2019. The combo chart shows the HIV testing and treatment cascade for the index partners, disaggregated by state and KP type. The number of index partners who were tested for HIV (orange) and the number of index partners who were HIV positive is shown in the graph (grey). The blue line graph depicts the percentage of HIV-positive index partners who were started on antiretroviral therapy, whereas the green bubbles depict HIV positivity rates among index partners according to KP typologies.

**Figure 4:**
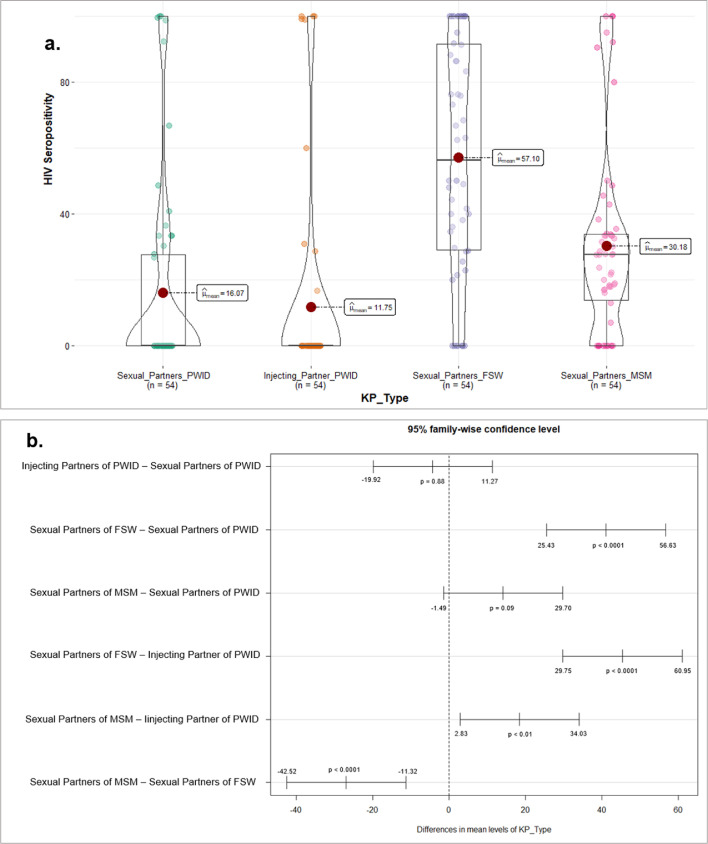
Distribution of HIV Seropositivity across KP types, October 2018 – September 2019 (a. top panel) and Tukey-HSD test-95% family-wise Confidence Level (b. bottom panel). In Figure 4a, the violin plots show the relationship of KP types to HIV seropositivity. The red dot represents the median, and the box plot elements show the median HIV seropositivity for injecting partners of PWID is lower than for other KP types. The horizontal bars in the center represents the interquartile range, and the other points are “outliers” using a method that is a function of the interquartile range.

In Akwa Ibom, 48.2% (3640/7556) of people received HIV testing and counseling, 32.6% (2462/7556) in Cross River, and 19.2% (1454/7556) in Lagos (Figure 3). HIV seropositivity rate was 89.1% (2194/2462) in Cross River, 31.9% (1162/3640) in Akwa Ibom and 27.3% (397/1454) in Lagos (Figure 3). In Cross River, HIV seropositivity was comparable in PWID injecting partners and PWID sexual partners (99.1%; 581/586 and 98.9%; 433/438) respectively. HIV seropositivity was significantly higher in MSM partners (95.6%; 605/633) than in FSW partners (71.4%; 575/805) in Cross River, z-test = 13.2, *p* < 0.00001. In Akwa Ibom HIV seropositivity was considerably higher in FSW partners (41.3%; 566/1371) than in MSM partners (25.2%; 490/1948), z-test = 9.8, *p* < 0.00001. HIV seropositivity among PWID sexual partners (50.0%; 2/4) and PWID injecting partners (47.5%; 19/40) in Lagos was not statistically different, z-test = 0.09, *p* = 0.928 (Figure 3). The mean HIV seropositivity was 57.1% among FSW partners, 30.2% among MSM partners, 16.1% among PWID sexual partners, and 11.7% among PWID injecting partners (Figure 4a). We found a statistically significant difference in mean HIV seropositivity by KP type (f(3)=23.07, *p* < 0.001). In Figure 4b, The Tukey-HSD test showed that HIV seropositivity rates among FSW partners were substantially higher on average than HIV seropositivity rates among sexual and injecting partners of PWID. The mean HIV seropositivity difference between MSM partners and injecting PWID partners is 18.4, with MSM partners averaging 18.4 points higher. HIV seropositivity was considerably higher among MSM partners than among injecting partners of PWID and sexual partners of FSW, respectively. In Lagos, almost all partners were linked to ART. Cross river showed similar results. Linkage to ART was lower than the UNAIDS-second 90 goal in Akwa Ibom across FSW, MSM, and PWID sexual partners (Figure 3).

## Discussion

Our study describes the implementation of a community-led index partner testing cascade as part of the national KP program. While the proportion of KP index clients who accepted PN or index testing services was low (29.7%), HIV testing uptake among partners of index clients was high (84.1%). It's worth noting that there's a scarcity of data on HIV index partner testing acceptance rates, particularly among KP. According to a retrospective analysis of program data on index partner testing cascade among the general population in Zambia and Kenya, 93% and 98% of PLHIV, respectively, accepted partner notification services.^17^ Another study in Lesotho found that 75 percent of index clients who were offered index partner testing accepted it. When compared to the acceptability rate reported in the general population, the acceptance rate for index partner testing was low in our study. KP's continued stigma and discrimination because of their perceived health status, sexual orientation, and/or gender identity may have influenced index partner uptake.^18^ Clients' fear of stigma, discrimination, and other human rights violations may explain the low index partner acceptance rate in our study. According to recent research, fear of stigma and criminalization under the Same-Sex Marriage Prohibition Act, as well as discrimination against KP, are the major reasons for inadequate access to health care and limited participation of KP in national surveys. The Nigerian government increased the penalty for homosexuality to 14 years in prison in 2014. Anyone who helps gay couples might face a ten-year jail sentence.^25^ These kinds of laws that criminalize homosexuality have forced MSM into hiding, rendering them more vulnerable to HIV.^26^ Further studies are recommended to determine the immediate effect of this prohibitive act on stigma, discrimination, and engagement among MSM in HIV prevention and treatment services in Nigeria.

The proportion of the KP index partners tested were mostly males (79%) than females (21%). FSW and MSM's partners were more likely males, which led to the high number of male partners in our study compared to females. Testing uptake among men was therefore remarkably higher than in a previous study in Malawi and Rwanda, which found that nearly 70% of adult HIV tests reported in 76 low- and middle-income countries were among females.^15^ The approach also proved to be effective; half (49.7%) of the successfully referred contacts were found HIV positive, and of these, all were newly diagnosed or first-time testers. Available evidence suggests that PLHIV are mostly willing to disclose their HIV status and to participate in provider-assisted HIV partner notification.^27^ In our study, partner notification services were predominantly done through provider referral (68.3%) compared to client referral (30.1%). A meta-analysis of three individually randomized trials using all identified partners as the denominator showed that assisted partner notification services resulted in a 1.5-fold increase in the uptake of HTS among partners compared with passive referral.^28^ These results align with our study which indicated that provider referral resulted in a 2.3-fold increase in the uptake of HTS among partners compared with the passive referral. In a study by Gabriel et al.,^29^ researchers examined the acceptability of anonymous notification by a health service provider (i.e., provider referral) in a sample of incarcerated PLHIV, most of whom were sexually active and using drugs before incarceration. The study found that two-thirds (66.4%) of PLHIV endorsed provider referral as an acceptable method to notify their sex partners and nearly three-quarters (72.4%) endorsed provider referral to notify their drug-injecting partners. HIV testing uptake among partners of index clients elicited for HTS was substantially high in our study (84%).

The 49.7% HIV positivity rate in our study is consistent with a partner notification study conducted by Nguyen et al.,^30^ in which the positivity rate among partners of index clients was 41.9%. We observed that the HIV seropositivity in sexual (71%) and injecting (91%) partners of PWID was considerably higher than in partners of FSW (71.4%) and partners of MSM (37%). The high HIV seropositivity among partners of PWID is in line with the latest UNAIDS 2018 report, which showed that the risk of HIV acquisition among people who inject drugs was 22 times higher than in the general population.^31^ This risk derives primarily from sharing needles and injection equipment but is compounded through criminalization, marginalization, and poverty. The disproportionately high HIV seropositivity among PWID could be linked to the HIV infection dynamics among PWID that suggests overlapping risk groups with multiple transmission routes. For example, some PWID are sex workers, or buy or trade drugs for sex, or are MSM, and may gain HIV through sexual and injecting routes.^32^ The high seropositivity of HIV among FSW partners compared with MSM partners may indicate that sex work and/or transactional sex continues to be the most important cause of new HIV infection in Nigeria.^33^^,^^34^ According to UNAIDS multi-country analysis on new HIV infections by mode of transmission, about 10% of new infections are the result of sex work. The study indicated that there would be about one and a half times more new infections due to sex work if it were not for the high levels of condom use reported in sex worker contacts.^35^

In Sub-Saharan Africa, it is believed that more than half (55%) of all sex workers have HIV.^36^ Even though sex workers are disproportionately impacted by HIV in every country in East and Southern Africa, HIV prevalence among this community varies widely, ranging from 5.5 percent in Madagascar to more than 70 percent in Lesotho and Uganda. HIV prevalence among sex workers in Nigeria was above 30% in 2007.^37^ There is no federal or national legislation prohibiting sex work in Nigeria. Although sex work is illegal in Nigeria's northern states due to Sharia Law. Sex work is legal in all of Nigeria's Western, Eastern, and Southern states. As a result, in such jurisdictions, police or security services are unable to apprehend sex workers.^38^ The HIV positivity rate among FSWs in our research was comparable to that of countries such as Botswana, Malawi, Rwanda, and Zimbabwe, where more than 40% of female sex workers are living with HIV.^39^ It is estimated that at least 90% of sex workers in East and Southern Africa are female, although selling sex is also common among men who have sex with men.^40^ HIV positivity in our study was higher among males than females. A recent report from UNAIDS shows that in sub-Saharan Africa, men and boys living with HIV are 20% less likely than women and girls living with HIV to know their HIV status, and 27% less likely to be accessing treatment.^41^ In western and central Africa, only 25% of men living with HIV are accessing treatment.^18^ When people are not on treatment, they are more likely to transmit HIV. The report also shows that HIV prevalence is consistently higher among men within key populations, and outside of eastern and southern Africa, 60% of all new HIV infections among adults are among men.

Condom usage by sex workers and their clients varies greatly. In some cases, sex workers have no access to condoms, have trouble negotiating their use with clients, or are unaware of their importance. The use of condoms by sex workers and their clients varies significantly. Sex workers may lack access to condoms, have difficulty negotiating their usage with clients, or are uninformed of their significance. In other situations, authorities seize or destroy condoms used by sex workers. Physical and sexual assault and harassment of sex workers carrying condoms were revealed in a 2012 study in Kenya, South Africa, and Zimbabwe. The fear of arrest for condom possession was also being used by police to blackmail and abuse sex workers.^42^ Emily et al. performed a qualitative study in 2019 to better understand the nature and consequences of GBV among FSW, MSM, and Transgender women, to influence HIV policy and programming and protect KPs' human rights. The study found that emotional and economic GBV were the most reported, but that sexual and physical GBV, as well as other human rights violations, were reported by about three-quarters of participants.^43^ GBV occurred most often at home, in places where sex work took places such as brothels, bars, and on the street; public areas such as parks, streets, and public transportation; health care facilities, police stations; and religious settings and schools for transgender women and MSM. To reduce the national burden of HIV while also promoting key populations' human rights, the national HIV program implements a variety of diversified preventive and treatment package interventions that address both HIV and GBV at the community and OSS levels. Modeling estimates in Kenya show that a reduction of approximately 25% of HIV infections among sex workers may be achieved when physical or sexual violence is reduced.^44^ In Uganda, a mixed-methods study investigated the knowledge and attitudes of FSWs and truckers about condom use. Condom awareness was high, with 97% of FSWs and 95% of truckers agreeing that "using condoms properly and regularly decreases the risk of HIV transmission." Condom usage was largely regarded positively, with 91 percent of FSWs and 82 percent of truckers believing that “condom use is the best means of HIV prevention.” Poverty, male partners' unwillingness to use condoms, alcohol usage before sex, and perceptions that condoms "kill the mood for sex" are all obstacles to regular condom use, according to qualitative findings.^21^

The national HIV/AIDS program provides targeted HIV testing services to KP and their high-risk contacts. During the study period, the following HIV testing modalities were implemented: KP testing in mobile or temporary testing locations, such as community centers, schools, workplaces, hotels, clubs, tents, and vans; voluntary counseling and testing which includes testing in voluntary counseling and testing centers outside of a health facility (i.e., “one-stop shops”), and index partner testing. According to the national KP program, HIV seropositivity or HIV positivity yield (the percentage of positives found out of those who were tested and received their test results) varies disproportionately across the testing modalities. Overall, HIV positivity yield among KP in our study was 49.7%. According to the national KP program, this was 9.6% in the mobile testing modality and 10.7% in the voluntary counseling and testing modality (see Supplementary 2). These results suggest that index testing model can be used to optimize HIV case identification efforts compared to the other testing models. According to recent research in Lesotho, the HIV index testing model had a statistically significant higher HIV positivity yield among adult clients tested than other HIV testing models (17.6% vs. 5.6 percent, *p* = 0.0009).^45^

Provision of partner notification services in the US and Europe is safe and effective, as well as cost-effective for HIV case-finding and linkage to care.^46^^,^^47^ Overall, our study shows high ART linkage rate (93.0%) with the HIV index testing model. Before the launch of PN services in 2017, overall linkage to ART among KP was marginally above half (53.7%) in Nigeria. Between October 2017 and September 2019, the linkage to ART increased by thirty-nine percentage points. There is a remarkably high linkage rate among FSW, MSM, and PWID sexual and injecting partners in all states, which confirms findings from earlier studies in other settings. Research done in Vietnam to explore assisted partner notification services as part of community testing services for key populations reported that 97.7% of sexual partners of index clients-initiated ART during the study period.^19^ The research done in Kenya and South Africa demonstrated that a family-centered model leads to more success in finding HIV positives and linking them to care and treatment.^48^^,^^49^ The high enrolment in care and high uptake of treatment services can be attributed to a more personal engagement of sexual and injecting partners by HTS providers and peer-outreach workers at the community level.

A major strength of this study was the large sample size of 7556 partners from index KP, disaggregated by FSW, MSM, and PWID. The large sample of KP data collected and used in our study compared to other previous studies can be attributed to the peer outreach model. In this model, KPs are trained as peer-outreach workers to increase demand for tailored HIV services, improve the quality of behavior change communication and increase access to HIV testing services, the starting point of the key population cascade, via social networks.

A key limitation of our study was that program data used for this analysis did not include demographic, socio-economic, and/or clinical characteristics of the study population that could provide more insights into the study population's risk behavior and the reasons for the variability of HIV seropositivity. We only had quantitative data and did not collect qualitative data that would have helped determine preferred referral strategies by index clients for partner notification. Consequently, other important variables were not available for analysis, particularly the age of sexual and injecting partners and the HIV status of serodiscordant couples. The national KP program does not break down HTS or index testing services by venue, which would have provided information on the proportion of index partner testing done at each location. Another limitation was the absence of data on viral suppression, which would have given information on the status of the last UNAIDS goal.

Combining passive and assisted HIV PN services as part of community-led HTS may help improve HIV case-finding approach for KP and contribute to reaching male partners of KP. Offering partner notification services from existing community settings (e.g., One-Stop Shops ART clinics) could greatly expand access to testing and linkage to care and treatment among people at very high risk of HIV infection, with limited additional burden on the health system. Allowing KP index clients to choose their preferred referral method may have resulted in greater uptake of the referral process, resulting in more partners of index clients being elicited for HIV testing. Our findings suggest that offering index clients options for passive or provider-facilitated notification and referral may result in a high uptake of PN services. Further research is needed to evaluate whether partner notification strategies, tailored differently, could be more successful in reaching multiple sexual and injecting partners of KP.

## Declaration of interests

In this study, we report no financial or non-financial competing interests. OK was supported by a professorship grant from the Swiss National Science Foundation (Grant no. 163878) and a Swiss National Science Foundation project Grant (320030_192452). All other authors report no disclosures.

## Appendix Supplementary materials

Image, application 1

## References

[1] Scribd. (n.d.). *Prevention gap report - 2016 - UNAIDS*. Scribd. Retrieved October 18, 2021, from https://pt.scribd.com/document/338616519/Prevention-Gap-Report-2016-UNAIDS.

[2] World Health Organization (2015). https://www.scirp.org/(S(351jmbntvnsjt1aadkposzje))/reference/referencespapers.aspx?referenceid=2238595.

[3] Organization, W.H. (1970, January 1). Consolidated guidelines on HIV prevention, diagnosis, treatment, and care for key populations. Pesquisa. Retrieved October 18, 2021, from https://pesquisa.bvsalud.org/portal/resource/en/biblio-911405.

[4] (HTS_Index) number of individuals who were identified and tested using index testing services and received their results. (HTS_Index) Number of individuals who were identified and tested using Index testing services and received their results | Indicator Registry. (n.d.). Retrieved October 17, 2021, from https://indicatorregistry.unaids.org/indicator/htsindex-number-individuals-who-were-identified-and-tested-using-index-testing-services.

[5] https://www.usaid.gov/global-health/health-areas/hiv-and-aids/technical-areas/key-populations

[6] Merrigan M., Azeez A., Afolabi B. (2011). HIV prevalence and risk behaviours among men having sex with men in Nigeria. Sex Transm Infect.

[7] Top nav. High HIV prevalence among men who have sex with men in Nigeria: Implications for combination prevention, Population Council. (n.d.). https://www.popcouncil.org/research/high-hiv-prevalence-among-men-who-have-sex-with-men-in-nigeria-implications. 10.1097/QAI.0b013e31828a3e60 23406978

[8] Mason K., Ketende S., Peitzmeier S., Johns Hopkins Bloomberg School of Public Health (2013). https://www.liebertpub.com/doi/10.1089/aid.2013.0092.

[9] Vuylsteke B., Semde G., Sika L. (2012). High prevalence of HIV and sexually transmitted infections among male sex workers in Abidjan, côte d'ivoire: need for services tailored to their needs. Sex Transm Infect.

[10] Sheehy M., Tun W., Vu L., Adebajo S., Obianwu O., Karlyn A. (2014). High levels of bisexual behavior and factors associated with bisexual behavior among men having sex with men (MSM) in Nigeria. AIDS Care.

[11] (2020). CROI Conference.

[12] Onovo A., Kalaiwo A., Katbi M., Ogorry O., Jaquet A., Keiser O. (2021). Directory of open access journals. JMIR Public Health Surveill.

[13] National HIV&AIDS and ReproductiveHealth Survey. (n.d.). https://naca.gov.ng/wp-content/uploads/2016/11/NARHS-Plus-2012-Final-18112013.pdf.

[14] Larsson E.C., Okong P., Thorson A., Ekström A.M. (2007). Antiretroviral treatment of HIV in Uganda: a comparison of three different delivery models in a single hospital. Trans R Soc Trop Med Hyg.

[15] Demographic patterns of HIV testing uptake in Sub-Saharan ... (n.d.). Retrieved October 18, 2021, from https://dhsprogram.com/pubs/pdf/CR30/CR30.pdf.

[16] Index case testing: A promising strategy for achieving HIV ... (n.d.). Retrieved October 18, 2021, from https://www.msh.org/sites/msh.org/files/cdc_-_index_case_brief.pdf.

[17] Matovu J.K., Ssebadduka N.B. (2013). Knowledge, attitudes & barriers to condom use among female sex workers and truck drivers in Uganda: a mixed-methods study. Afr Health Sci.

[18] U.S. National Library of Medicine (2021). Hiv partner notification services. Guidel HIV Self Test Partner Notif Suppl Consol Guidel HIV Test Ser.

[19] Decker M.R., Wirtz A.L., Pretorius C. (2016). https://jhu.pure.elsevier.com/en/publications/estimating-the-impact-of-reducing-violence-against-female-sex-wor-4.

[20] Evens E., Lanham M., Santi K. (2019). Experiences of gender-based violence among female sex workers, men who have sex with men, and transgender women in Latin America and the Caribbean: a qualitative study to inform HIV programming. BMC Int Health Hum Rights.

[21] Www.unaids.org. (n.d.). Retrieved October 18, 2021, from https://www.unaids.org/sites/default/files/media_asset/201506_JC2743_Understanding_FastTrack_en.pdf.

[22] “Nigeria_GARPR_2015_Report.”*NACA Nigeria*, 14 Nov. 2016, naca.gov.ng/nigeria_garpr_2015_report/.

[23] The African Commission (2017) ‘HIV, the law and human rights’ [pdf]

[24] Ochako R., Okal J., Kimetu S., Askew I., Temmerman M. (2021). Female sex workers experiences of using contraceptive methods: a qualitative study in Kenya. BMC Womens Health.

[25] NIGERIA indicator and technical Impact survey report. (n.d.). Retrieved September 12, 2021, from http://ciheb.org/media/SOM/Microsites/CIHEB/documents/NAIIS-Report-2018.pdf.

[26] Projecting the demographic consequences of adult HIV*...* (n.d.). Retrieved September 12, 2021, from https://sti.bmj.com/content/sextrans/80/suppl_1/i14.full.pdf.

[27] Carla Makhlouf Obermeyer P.B. (2021, October 18). Facilitating HIV disclosure across diverse settings: a review. PubFacts.

[28] Passin W.F.; Kim A.S.; Hutchinson A.B.; Crepaz N.; Herbst J.H.; Lyles C.M.;; (n.d.). A systematic review of HIV partner counseling and referral services: client and provider attitudes, preferences, practices, and experiences. Sex Transm Dis. Retrieved October 18, 2021, from https://pubmed.ncbi.nlm.nih.gov/16505750/. 10.1097/01.olq.0000194597.16236.48 16505750

[29] Exploring the acceptability of HIV partner notification in*...s* (n.d.). Retrieved October 18, 2021, from https://pdfs.semanticscholar.org/400c/1c3978d9064706eddb45855778c2bdf9e6cb.pdf

[30] Nguyen V.T.T., Phan H.T.T., Kato M. (2019). Community-led HIV testing services including HIV self-testing and assisted partner notification services in Vietnam: lessons from a pilot study in a concentrated epidemic setting. Wiley Online Library.

[31] *Miles to go – closing gaps; breaking barriers; righting injustices - world*. ReliefWeb. (n.d.). Retrieved October 18, 2021, from https://reliefweb.int/report/world/miles-go-closing-gaps-breaking-barriers-righting-injustices.

[32] *Overlapping substance using high-risk groups and ...* (n.d.). Retrieved October 18, 2021, from https://research-information.bris.ac.uk/en/publications/overlapping-substance-using-high-risk-groups-and-infectious-disea.

[33] Sheehy M., Tun W., Vu L., Adebajo S., Obianwu O., Karlyn A. (2021). High levels of bisexual behavior and factors associated with bisexual behavior among men having sex with men (MSM) in Nigeria. AIDS Care.

[34] Oldenburg C.E., Perez-Brumer A.G., Reisner S.L., Mimiaga M.J. (2015). Transactional sex and the HIV epidemic among men who have sex with men (MSM): results from a systematic review and meta-analysis. AIDS Behav.

[35] Www.unaids.org. (n.d.). Retrieved October 18, 2021, from http://www.unaids.org/sites/default/files/en/media/unaids/contentassets/documents/countryreport/2010/201003_MOT_West_Africa_en.pdf.

[36] MacAllister J., Sherwood J., Galjour J. (2015). A comprehensive review of available epidemiologic and HIV service data for female sex workers, men who have sex with men, and people who inject drugs in select west and Central African countries. J Acquir Immun Defic Syndr.

[37] Mountain, E., Mishra, S., Vickerman, P., Pickles, M., Gilks, C., & Boily, M.C. (n.d.). Antiretroviral therapy uptake, attrition, adherence and outcomes among HIV-infected female sex workers: a systematic review and meta-analysis. PLoS ONE. https://journals.plos.org/plosone/article?id=10.1371%2Fjournal.pone.0105645. 10.1371/journal.pone.0105645 PMC4179256 25265158

[38] Unaids (2019, September 5). Global AIDS UPDATE 2019 - communities at the centre. UNAIDS Eastern S Afr.

[39] Federal Ministry of Health (2010). https://www.scirp.org/reference/ReferencesPapers.aspx?ReferenceID=1206400.

[40] Umah O. (2019, May 5). PROSTITUTION is not a crime in all parts of Nigeria and police cannot Arrest PROSTITUTES.* daily LAW TIPS (Tip 324A) BY Onyekachi umah, ESQ., LLM. ACIARB(UK). Learn Niger Laws.

[41] AIDSinfo. (n.d.). https://aidsinfo.unaids.org/.

[42] (2020). HIV and Aids in east and Southern Africa regional overview. Avert.

[43] Unaids.org (2017). On world AIDS Day, UNAIDS warns that men are less likely to access HIV treatment and more likely to die of aids-related illnesses. UNAIDS.

[44] *Criminalizing condoms*. Open Society Foundations. (n.d.). https://www.opensocietyfoundations.org/publications/criminalizing-condoms.

[45] Jubilee M.;Park F.J.;Chipango K.;Pule K.;Machinda A.;Taruberekera N.; (n.d.). HIV index testing to improve HIV positivity rate and linkage to care and treatment of sexual partners, adolescents and children of PLHIV in Lesotho. PLoS ONE. Retrieved November 21, 2021, from https://pubmed.ncbi.nlm.nih.gov/30917167/. 10.1371/journal.pone.0212762 PMC6436679 30917167

[46] Hogben M. (1970). The effectiveness of HIV partner counseling and referral services in increasing identification of HIV-positive individuals: a systematic review. Database Abstr Rev Effect (DARE) Qual Asses Rev.

[47] From the Division of HIV/AIDS Prevention. (n.d.). *Cost-effectiveness of counseling and testing and partner...: AIDS*. LWW. Retrieved October 19, 2021, from https://journals.lww.com/aidsonline/Fulltext/1999/09100/Cost_effectiveness_of_counseling_and_testing_and.19.aspx.

[48] PMC, E. (n.d.). Europe PMC. Retrieved October 19, 2021, from https://europepmc.org/abstract/MED/22353553.

[49] PMC, E. (n.d.). Europe PMC. Retrieved October 19, 2021, from http://europepmc.org/abstract/MED/27547750.

