# Supplementary material for: Diagnosis and case finding according to key partner risk populations of people living with HIV in Nigeria: A retrospective analysis of community-led index partner testing services
Source: eClinicalMedicine. 2022 Jan 4;43:101265. doi: 10.1016/j.eclinm.2021.101265 (PMC8741428; doi:10.1016/j.eclinm.2021.101265)
Supplement: Supplementary file 1 [file mmc1.docx]

1. Appendix 1: Distribution of 2019 UNAIDS SPECTRUM estimate and study location. On the main map of Nigeria, blue boxes were utilized to highlight the study locations on the inset map on the right, which offers information on the three study areas. The main map depicts the distribution of PLHIV burden by state using the UNAIDS 2019 PLHIV estimates from SPECTRUM.


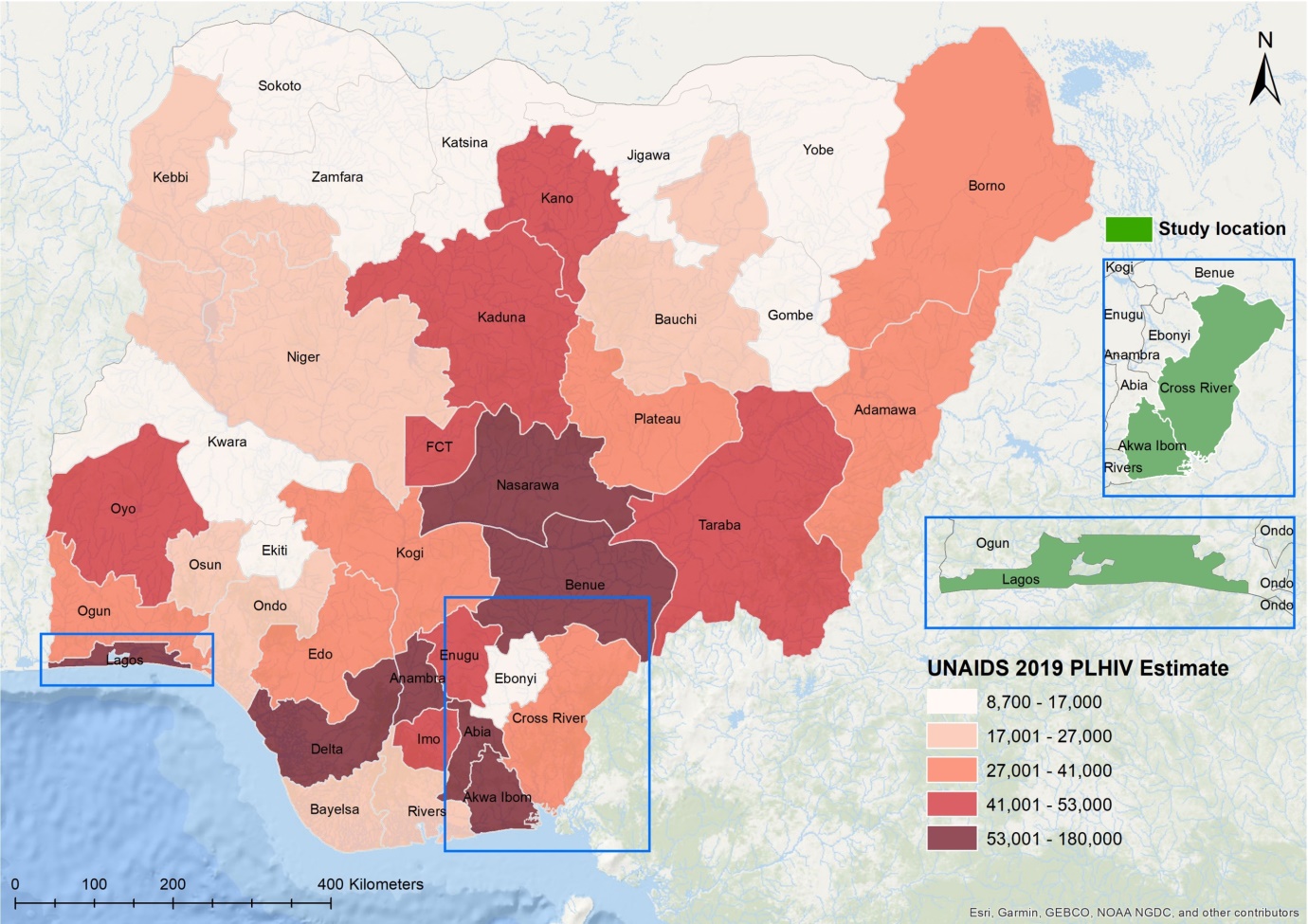


1. Appendix 2: Distribution of HIV Tests and HIV Positivity Yield by Testing Modalities, October 1, 2018 – September 30, 2019. The number of KP tested for HIV is shown by the green bars, the number of KP who tested HIV positive is represented by the red bars, and the testing yield is represented by the yellow bubbles.


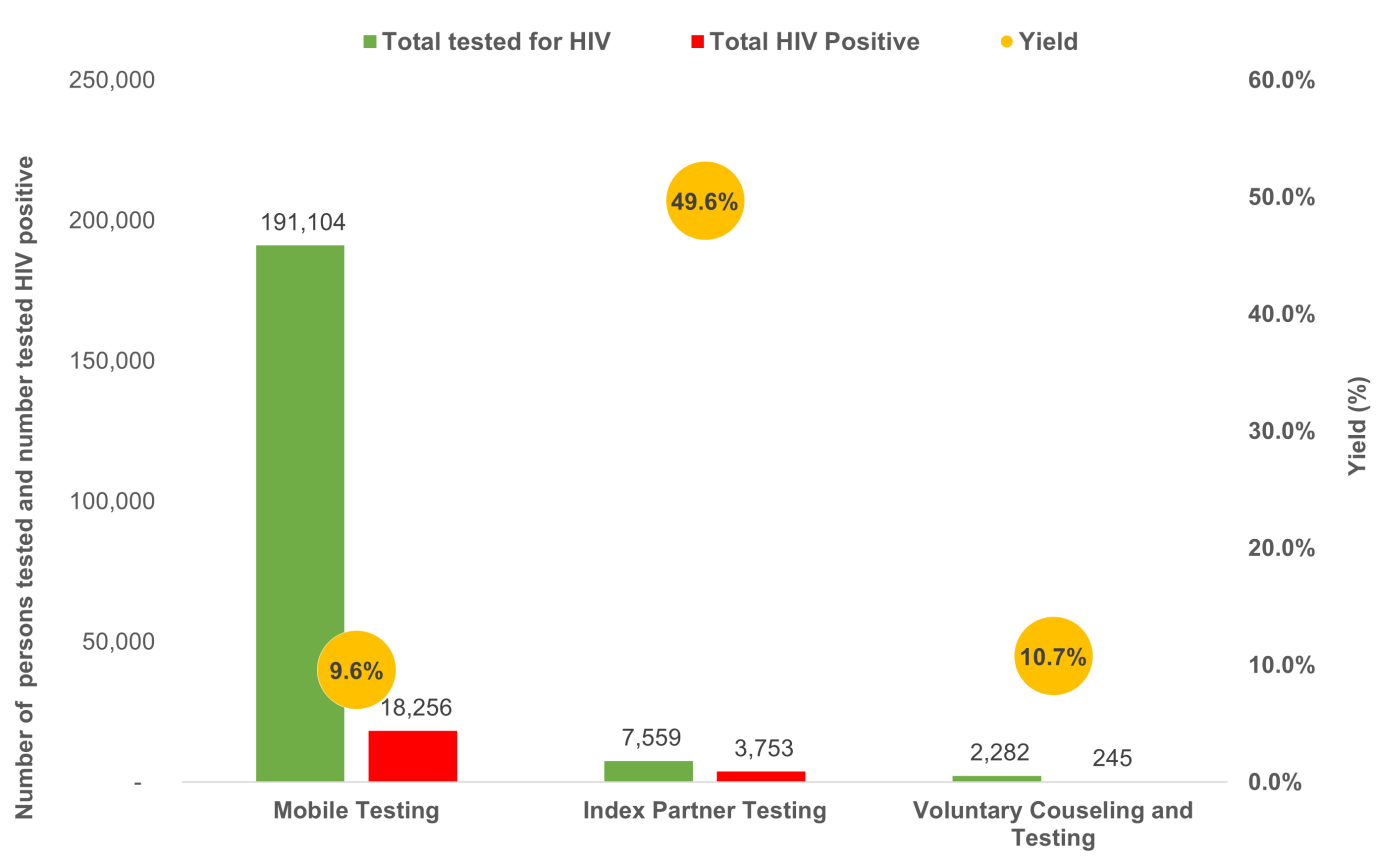


1. UNAIDS 2019 PLHIV estimates from SPECTRUM
